# How villagers in central Sierra Leone understand infection risks under threat of Covid-19

**DOI:** 10.1371/journal.pone.0235108

**Published:** 2020-06-24

**Authors:** Foday Mamoud Kamara, Esther Yei Mokuwa, Paul Richards

**Affiliations:** Njala University, Mokonde, Sierra Leone; Helen Keller International, SIERRA LEONE

## Abstract

**Background:**

Concern has been expressed over how well Africa is prepared to cope with the pandemic of Covid-19. Will rural populations with low levels of education know how to apply community-based infection control? We undertook fieldwork in two villages in central Sierra Leone to gain insight into how rural people faced with Covid-19 assess epidemic infection risks.

**Methods:**

Two communities were selected based on prior contrasted exposure to Ebola Virus Disease–one with substantial number of cases and the other having resisted infection through strong community sequestration measures. We assessed understanding of infection risks via an experimental game. This asked players to express a preference for one of two diseases, one resembling Ebola with lower risk of infection and the other resembling Covid-19 with lower risk of death. Players were not told the identity of the diseases.

**Results:**

In total 107 adult villagers played the game (58% women). Half (52%) preferred the disease model with lower risk of infection, 29% preferred the model with lower risk of death, while 21% saw the combined risk of infection and death as being equivalent. Differences in reactions between the two locations were small despite different experiences of Ebola. Asked to explain their choices 48% of players cited information on infection risks modelled by the game and 31% stated that their choices reflected awareness of the need for personal action and respect for local regulations. We concluded that villagers thoughtfully assess disease risks and that some are good intuitive statisticians.

**Conclusions:**

Results suggest rural people in Sierra Leone retain the lessons of experience from the Ebola outbreak of 2014–15 and will be able to apply these lessons to a new infectious disease for which have no prior practical experience. Our expectation is that rural populations will understand Covid-19 control measures, thus reducing need for draconian enforcement.

## Introduction

Concern has been expressed in media debate about the potential damage that could be caused by the spread of Covid-19 in Africa on account of weak health systems and lower levels of education. In particular, there has been some speculation about capacity to apply epidemiological concepts. Social distancing, for example, demands not only physical separation but also an appreciation of the need to reduce chances of social contacts.

To contribute to a better appreciation of these concerns this paper addresses the question of how well rural African populations understand and apply information relating to risks associated with infectious diseases. The data relate to rural populations in Sierra Leone exposed both to Ebola Virus Disease (EVD) in 2014–15 and now to Covid-19 in 2020.

Numerical ability among people without formal education has been the subject of some research enquiry by anthropologists and others. By and large these studies have demonstrated that alleged computational handicaps among non-literate populations were more apparent than real.

Gay and Cole found that non-literate rice farmers were better able to estimate volumes than US college graduates deployed by the Peace Corps to teach mathematics in Liberian schools [1]. Lave documented the truly astonishing capacities of tailors in Monrovia to cut garments to fit varying body sizes and shapes without use of patterns [2].

The emergence and species-wide distribution of basic statistical capacities is a topic addressed by evolutionary anthropologists. In an influential contribution, Cosmides and Tooby conclude that the view *most common in the literature on judgment under uncertainty—that our inductive reasoning mechanisms do not embody a calculus of probability—will have to be re-examined*. *From an ecological and evolutionary perspective*, *humans may turn out to be good intuitive statisticians after all* [3].

Broadly speaking, it has been assumed that popular decision-making regarding hazards is undertaken under uncertainty, guided by common-sense heuristics [4,5]. Here, we attempt to assess whether or not African villagers conceptualise decisions regarding infection in terms of risks. Our paper offers provisional findings on this topic, based on data gathered in two Ebola-affected rural communities in central Sierra Leone immediately prior to the 3-day lockdown for Covid-19 in the first week of April 2020.

## Methods and materials

We collected data using a rapid action research approach. Two communities were chosen because our research team was well-known in these locations through work on impact of EVD [6], and because we had ethical approval for ethnographic and survey work on pandemic preparedness provided by the Institutional Review Board of Njala University. This meant we could implement fieldwork quickly to provide information on how well Covid-19 prevention strategies were likely to work in these settings.

For data collection we used a simple game or test devised to encourage villagers to talk comparatively about infection risks. Each iteration of the game took about 15 minutes to complete, including time taken to explain the game, and for the person tested briefly to explain their choices.

In each village we first held a public consultation with the chief, explaining that the purpose of our visit was to run a small game, and seeking permission to proceed. We also explained that we would return to explain the results of the game when results were processed (see below). Sampling was opportunist. Research assistants (RAs) approached all adult male or female villagers present, explained the game and asked who would like to play. Willingness to play constituted informed consent. This was witnessed by the field supervisor and the community chiefs, since a majority of participants do not read or write.

For the larger of the two villages (both are here identified by pseudonyms) we have a baseline census undertaken in mid-2019 as part of a larger research project on pandemic preparedness, so were able to check the biases inherent in this mode of sampling. From this, we know the sample under-represents adult males (= 41%). Many of the absentee males were busy clearing rice farms at this time.

The two communities were contrasted in terms of their exposure to EVD in 2014–15. One village (Taninihun, popn. c. 250) had a substantial number of cases early in the 2014–15 epidemic [6]. The other community (Tawoveihun, popn. c. 850) was located in a chiefdom (lowest level of the hierarchy of local government administration) which prevented EVD infection throughout the 2014–15 epidemic through rigorously enforced community lockdown.

Both communities were exposed to information on Covid-19, mainly by radio broadcasting. At the date of writing (early May 2020) no cases had been reported as testing positive for Covid-19 in the two districts concerned (Bo and Moyamba). Altogether 72 iterations of the game were carried out in Tawoveihun and 35 iterations in Taninihun (labelled “village 2A” in [6]). This represents approximately one fifth of the total adult population of each village.

### The game

In every run of the game the researcher (a trained Mende-speaking research assistant) lays out two sets of twenty stones or pebbles in two ranks of ten stones. Each set of stones, represented by the leaf of an orange and a mango tree, two common fruit trees in every Mende village, represents one of two epidemic disease. These diseases are referred to throughout as “orange” and “mango”. Neither pattern of stones was identified explicitly as a proxy for Covid-19 or Ebola, though judging by the comments (see S1 Data) some players may have guessed the implied identities.

For each disease players are told the first rank of stones represented the chance of being infected and of not being infected. For “orange” this is 5 stones (infected) and 5 stones (not infected). For “mango” this is 1 stone (infected) and 9 stones (not infected). The second rank of ten stones represents the risk of dying from or surviving the disease. For “orange” this is 1 stone (death) and 9 stones (recovery), and for “mango” this is 5 stones (death) and 5 stones (recovery).

These values only roughly match the infection and death risks associated with Covid-19 and Ebola. More stones would have achieved a better match, but we judged that this might make the game more difficult for the players. Nevertheless, the two patterns capture a major contrast between the two diseases, that with EVD there are few infections but more of those infections prove deadly, and that with Covid-19 there are many infections, but fewer are deadly.

It will also be realised, from the set-up of the game, that we deliberately chose to set the combined probability of infection and death as the same in both cases (p = 0.05).

Again, this was not pointed out to villagers. Instead, RAs were instructed carefully to note down any player who reported this equivalence, but without commenting further, to ensure that if other players arrived at the same conclusion it was not because it had been endorsed by our field team within their hearing. Our aim was to find out if there was any intuitive grasp of the multiplication rule for independent probabilities.

Players were invited to explain which disease they thought was preferable, and why. Iterations of the game were documented in field notebooks against the name and gender of the player, together with each player’s brief comment on the game or their choices. Field notebooks were then photographed and sent for data analysis to the Principal Investigator in the Netherlands via WhatsApp (an encrypted connection). The anonymised spreadsheet version is made available in the S1 Data.

## Results

Overall (Table 1), there was a higher preference (52% of all responses) for “mango” (modelling EVD). Disease “orange” (modelling Covid-19) attracted just over a quarter (27%) of all responses. Players finding no difference between the two disease models accounted for 21% of all responses.

**Table 1:** Disease preferences in two villages in central Sierra Leone.

	Disease “orange”	Disease “mango”	Both are the same
Tawoveihun	16	35	21
Taninihun	13	21	01
Total	29 (27%)	56 (52%)	22 (21%)

Gender differences in preferences (Table 2) were not statistically significant.

**Table 2:** Disease preferences by gender (two villages in central Sierra Leone).

	“Orange”	“Mango”	TOTALS
Male	14	25	39
Female	17	31	48
TOTALS	31	56	87

Fisher exact test = 1.00, not significant at p<0.05.

In Tawoveihun, “orange” is chosen more often by women than by men (Table 3), but the gender difference is not statistically significant.

**Table 3:** Disease “preferences” by gender in Tawoveihun.

	Disease “orange”	Disease “mango”	TOTAL
Female	12	17	29
Male	5	18	23
TOTAL	17	35	56

Fisher exact test = 0.1521, not significant at p<0.05.

In Taninihun “mango” is chosen more often by women than men (Table 4). Again, the difference is not statistically significant.

**Table 4:** Disease preferences by gender in Taninihun.

	Disease “orange”	Disease “mango”	TOTAL
Female	5	14	19
Male	8	7	15
TOTAL	13	21	34

Fisher exact test = 0.1596, not significant at p<0.05.

Three kinds of responses (Table 5, 89% of all remarks) dominated comments after people made choices in the game–need for rules governing e.g. quarantine, comments on personal capacities to prevent or avoid infection (often made in the context of justifying a choice for disease “mango”), and comments about the relative risks to be inferred from “reading the stones” (i.e. recognition of a pattern in the way the stones were cast, as in divination practices).

**Table 5:** Frequency of types of comments villagers made in justifying their preferences.

	Comments on need for rules e.g. governing quarantine	Comments on prevention or avoidance of infection	Comments about chances learnt from the game	Other comments
Tawoveihun	12	23	48	13
Taninihun	04	24	23	3
TOTAL	16 (10,6%)	47 (31,1%)	72 (47,7%)	16 (10,6%)

Some persons contributed more than one comment.

The largest group of comments (48 per cent) related to what can be learnt from the game itself (Table 5). Within this set, 25 comments specifically mentioned the distribution of the stones in the game (S1 Data). The second largest group of comments (31 per cent) related to steps that can be taken to avoid or prevent infection (Table 5).

## Discussion

Ebola Virus Disease is a terrifying disease. Thus, it seems at first sight paradoxical that villagers should consistently express a preference for “mango”, the disease with a risk profile in the game similar to EVD. This preference is consistent across the two sites and genders.

Our suggested explanation relates to recent experience. Ebola had a low risk of spread once infection control measures were stringently applied. This was well understood in both villages. In Tawoveihun the chiefs led a community-based quarantine initiative and no cases occurred. In Taninihun there was a devastating outbreak caused by a funeral for a respected Islamic teacher as a result of which many of his pupils died. Enforced quarantine was a very painful experience, but people later realised this action had ended the outbreak. It seems clear from comments given to explain preferences that prospects for infection control had considerable weight. Choices were related to preventive measures or local byelaws in 42 per cent of comments (Table 5 and S1 Data). One player from Tawoveihun made the link between community action and avoidance of infection explicit by commenting that with Covid-19 “we will survive through *rules and regulations* like we did during the Ebola crisis” (Female, Tawoveihun, our emphasis).

Perhaps disease preferences also reflected lack of confidence in the possibility of cure. Neither local nor hospital treatments were effective for EVD. At the outset, only 30 per cent or so patients recovered, and even in the later stages, survival was hardly better than 60 per cent. Whether someone sick with the disease lived or died was more a matter for God. For this reason, it may have seemed better to pick the option with the lower risk of infection in the first place and stick to the rules.

Nevertheless, a considerable minority (27 per cent) preferred disease “orange” (with a Covid-19-like profile). These players mention the lower death rate. Some appear to have had the evident awfulness of death from Ebola in mind when rejecting the “mango” option. One man in Tawoveihun rationalised his choice for disease “orange” with the comment that “some sicknesses are worse than death” (S1 Data).

Just over one fifth of players (21 per cent) considered that there was no difference in ultimate outcome from the two disease profiles. Comments included that “the diseases are the same” (Female, Taninihun), “[both] seem the same and both are bad, except God helps you” (Female, Tawoveihun) and “I fear both because of the poverty of Africa” (Male, Tawoveihun).

A large number of comments suggested that players were taking specific note of information conveyed in the distribution of the stones. Some players were explicit in relating their preferences to numerical weights—for example “nine chances of not being infected” (S1 Data).

If there were intuitive probabilists among the players of the game we expect these to be among the 21 per cent who decided (correctly) that the combined probabilities of infection and death were the same in both models. This suggests a grasp of the multiplication rule for probability, even though players lacked much (or any) formal schooling.

We did not collect data on educational levels for players in this game, but census work for a larger project of which this study is a part tells us that older male farmers and village women rarely have more than a year or two of school education and many have no schooling whatsoever. Their number skills come from what Lave [2] describes as situated learning–i.e. acquired through practical experience in daily life.

It has been suggested that situated learning often does not transfer outside its immediate context–that judgments cannot be shifted between contexts. Our data, especially the comments of those who saw the two variants of the game having the same risk of eventual death, suggest the contrary, and that the two diseases–one known from experience, the other (as yet) only from reports–were handled according to a single calculus of risk.

Response to the game is an important part of what we set out to discover. Players of the game adapted to it with apparent ease, perhaps because talking about problems in this way reflects experience in village divination sessions, often conducted with pebbles, cowrie shells or nuts. Several informants reported their decisions in terms of information conveyed by the stones, an idiom familiar from the process of village divination [7]. In these cases, explanation of choices was phrased in language such as “the stones have already told me how I need to protect myself”. One woman noted that the stones “are talking about death” in both versions of the game.

These results suggest that games modelled on divinatory practices could provide public health practitioners with a generally useful means of conveying information about infection risks.

The game also provides a specific context to engage further in comparative discussion of infection risks associated with Covid-19. There has been widespread initial fear in rural Sierra Leone that Covid-19 is another Ebola. For effective response, the differences between EVD and Covid-19 need to be more fully explored. Specifically, we are in the process of returning to the two communities to discuss the implications of the results reported above. We then plan to use the game to set up discussion of variations in risks of Covid-19 by age.

We intend also to follow up on comments by players that require further contextualization. One of the most interesting (made by two people) is that “no one will swear on sickness”. Village disputes often are resolved by asking a person to swear publicly to the truth of their evidence. No one can be sure whether or not the swearer is a brazen liar. But sickness, it is suggested, is not something that can be evaded by bluster; risks of infection are real.

Overall, our evidence suggests that rural people in Sierra Leone respond to infection risks in rational and calibrated ways. The benefits of this response were experienced in the Ebola epidemic of 2014–15 [8]. We see no evidence that the same will not also be the case with Covid-19. Communities should be trusted to play a fuller part in infection control.

## Supporting information

S1 Data (XLSX)
